# Modulating T Cell Responses by Targeting CD3

**DOI:** 10.3390/cancers15041189

**Published:** 2023-02-13

**Authors:** Ashwathi Puravankara Menon, Beatriz Moreno, Daniel Meraviglia-Crivelli, Francesca Nonatelli, Helena Villanueva, Martin Barainka, Angelina Zheleva, Hisse M. van Santen, Fernando Pastor

**Affiliations:** 1 Molecular Therapeutics Program, Center for Applied Medical Research, CIMA, University of Navarra, 31008 Pamplona, Spain; 2 Instituto de Investigación Sanitaria de Navarra (IDISNA), Recinto de Complejo Hospitalario de Navarra, 31008 Pamplona, Spain; 3 Unidad Desarrollo y Función del Sistema Inmunitario, Centro de Biología Molecular Severo Ochoa, Consejo Superior de Investigaciones Científicas, Universidad Autónoma de Madrid, 28049 Madrid, Spain

**Keywords:** CD3, TCR, T cell engager, cancer immunotherapy, T cell modulation, antibodies, aptamers

## Abstract

**Simple Summary:**

CD3 complex provides the first signal sensed by the TCR of the lymphocyte to trigger its activation. Thus, it becomes a very attractive receptor to determine the fate of the immune response in different contexts from tolerance induction to immune activation. We discuss CD3-TCR complex assembly and the current and emerging approaches to harvest CD3 activity for immunotherapy.

**Abstract:**

Harnessing the immune system to fight cancer has become a reality with the clinical success of immune-checkpoint blockade (ICB) antibodies against PD(L)-1 and CTLA-4. However, not all cancer patients respond to ICB. Thus, there is a need to modulate the immune system through alternative strategies for improving clinical responses to ICB. The CD3-T cell receptor (TCR) is the canonical receptor complex on T cells. It provides the “first signal” that initiates T cell activation and determines the specificity of the immune response. The TCR confers the binding specificity whilst the CD3 subunits facilitate signal transduction necessary for T cell activation. While the mechanisms through which antigen sensing and signal transduction occur in the CD3–TCR complex are still under debate, recent revelations regarding the intricate 3D structure of the CD3–TCR complex might open the possibility of modulating its activity by designing targeted drugs and tools, including aptamers. In this review, we summarize the basis of CD3–TCR complex assembly and survey the clinical and preclinical therapeutic tools available to modulate CD3–TCR function for potentiating cancer immunotherapy.

## 1. Mobilizing the Immune Response in Context of Different Immunotherapeutic Strategies

One of the most successful breakthroughs in the fight against cancer has emerged in the past few decades with the advent of cancer immunotherapy [1,2]. Cancer vaccines, immune checkpoint blockade, and adoptive cell transfer therapies have revolutionized the treatment paradigm and standard of care protocols for treating cancers in the past few years [2]. They have shown tremendous success in the clinic, improving survival and quality of life for patients that would otherwise reach end of care with conventional therapies [2]. 

However, several challenges still need to be overcome to optimize cancer immunotherapy. The high tumor heterogeneity and intrinsic genetic and epigenetic variability of cancer make immunotherapy responses hard to predict and nonhomogenous [3]. Only a subset of patients responds to current immunotherapy approaches, and sometimes “secondary immune escape” occurs, causing relapse in patients who are currently in remission after successful initial cancer immunotherapy treatments [4]. 

Identification of tumor-immune molecular drivers might open the possibility of exploring alternative therapeutic strategies to enhance clinical response rates in cancer patients. Among the multiple factors that could determine the fate of the immune response in the context of cancer is the source and quality of tumor antigens [5]. 

Tumor antigenicity is a random process restricted by the frequency and type of tumor mutation in the cancerous lesion, and it is actively reshaped by immunoediting forces that restrict the expression of the most potent tumor antigens [6]. Generally, most tumors display limited tumor antigenicity. Therefore, it is essential to develop therapeutic approaches to bypass this lack of tumor antigenicity and increase “visibility” to the antitumor immune response [5]. Tuning the TCR/CD3 interaction, known as the first type of signal needed for T cell activation, is a strategic approach that may be used to achieve this goal. 

## 2. The T Cell Receptor: Intercepting Signals for T Cell Activation

### 2.1. Structure of the TCR/CD3 Complex

Incumbent to the function of T cells is the T cell antigen receptor complex or the TCR, a multimeric surface receptor that receives, integrates, and transduces the major histocompatibility complex (MHC)-restricted peptide antigen-based signals that are needed for activation of a T cell [7]. The TCR is made up of the α and β TCR chains that recognize the peptide–MHC complex. The CD3 signaling complex proteins are made up of δ-ε and γ-ε heterodimers that contain extracellular and intracellular domains, and a ζ- ζ homodimer that has a very short extracellular domain and a long intracellular domain. In an α-β T cell, the TCR is composed of a 1:1:1:1 ratio TCRαβ:CD3γε:CD3δε:CD3ζζ subunits [7,8,9]. Early mutagenesis and immunoprecipitation studies have provided us with knowledge of the precise stoichiometric composition of the TCR subunits [8] using digitonin-lysed T cell products or by artificially constructing mock T cells by transfection with the various CD3 subunits. It was not until 2019 that the extracellular and transmembrane structure of the human TCR/CD3 in its expressed unligated state was resolved using cryoelectron microscopy techniques [7]. Recently, the cryoEM resolved structure of a peptide–MHC (pMHC)-CD3–TCR complex was reported, showing, in line with previous findings, no major adjustments in the TCRαβ domain, but neither in the CD3 extracellular domains upon pMHC ligation [7,10]. 

#### 2.1.1. TCR Chains

**Figure 1:**
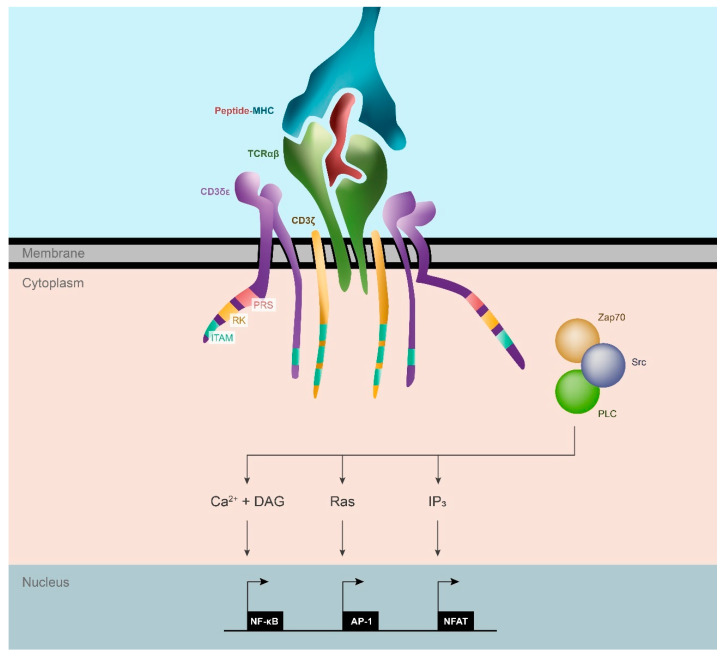
TCR structure and T cell activation pathway. The TCRαβ recognizes the pMHC complex on the antigen-presenting cell at the immunological synapse. The transduction of the signal is mediated by the ITAM domains located in the CD3 intracellular domains, which are phosphorylated by *Src* family kinases. The signaling axis comprises numerous factors, such as Zap70 and PLC. The cascade ends up with the production of second messengers (Ca^2+^, diacylglycerol (DAG), and IP_3_) and the activation of Ras. These mediators lead to the upregulation of gene transcription by NF-κB, AP-1, and NFAT, triggering the activation of the T cell.

The highly variable TCR α β heterodimer ligates with cognate pMHC (Figure 1). It intercepts the antigenic signal of activation but cannot initiate T cell signaling by itself because of the short cytoplasmic tails of each of the two chains. These short tails are devoid of immunoreceptor tyrosine-based activation motifs (ITAMs) whose phosphorylation by Src family kinases such as LCK and FYN triggers T cell activation [11].

TCR α and TCR β chains are glycoproteins consisting of two immunoglobulin domains each and are linked covalently by a disulfide bond. The evolutionary conserved acidic, negatively charged, amino acid residues in their transmembrane domains form ionic interactions with basic (positively charged) residues located in the transmembrane domains of the CD3 subunits [7,8]. The α chain shares homology with the light chain of an antibody, and the β chain with the heavy chain [12]. Each subunit consists of a constant region (proximal to the membrane) and a variable region (distal to the membrane) [7,12]. TCR α and TCR β genes are randomly assembled from highly diverse V, D (only TCRβ), and J gene segments and the constant gene segment via a RAG1/2 recombinases-dependent process. 

The V regions of each of the chains encode two of the three complementarity-determining regions (CDRs), whereas the third and most variable CDR is formed by random joining of the V (D) and J segments, with removal and nontemplated addition of nucleotides increasing diversity even further, thereby allowing for diverse antigen recognition by TCRs [13]. These hypervariable CDR loops bind to the MHC complexes presenting processed antigenic peptides (usually an 8 to 10 for MHC class I molecules and peptides of up to 20 aa for MHC class II molecules), and provide each TCR with its unique specificity, making each T cell different from its counterpart [13]. Thus, the immune system assembles a large, formidable army consisting of millions of T cells, each with a unique TCR, capable of recognizing and responding to millions of antigens specific to a wide range of pathogens as well as neoplastic tumor antigens [14,15]. 

#### 2.1.2. CD3 Subunits

The relay of signals received by the TCR chains into the cytoplasm is essential for T cell activation. As the TCR α and β chains lack known intracellular signaling motifs, the transduction of activation signals to second messengers and intracellular transcription factors is coordinated by the intracellular signaling competent partners of the TCR: the CD3 protein complex (Figure 1) [7].

CD3 is a multimeric protein complex comprising three different subunits: CD3δε, CD3γε, and CD3ζζ dimers. Homologous CD3δε and CD3γε are heterodimers with single extracellular immunoglobulin domains that interact with the TCR α and β chains and have relatively short intracellular signaling domains. CD3γε and CD3δε dimers are similar to the CD79αβ subunits of the B cell receptor (BCR), which serve as auxiliary signaling components of the BCR [16]. 

The ζζ homodimer has a negligibly short ectodomain but an elaborate cytoplasmic domain that is essential for T cell signaling and activation. Unlike the TCR α and β chains that are highly diverse and vary among T cells, the CD3 subunits are invariant elements, shared among all α-β T Cells [17]. 

The various CD3 subunits and their respective stoichiometries in human and murine T cells were identified through immunoprecipitation studies conducted in the 1990s [18,19,20,21]. Several of these fundamental findings still define current knowledge about the CD3/TCR: (1) the CD3 subunit is essential for T cell activation [22,23]; (2) the CD3/TCR complex works as one complete functional unit [7]; (3) each TCR/CD3 complex consists of a fixed stoichiometry of subunits and is suborganized as pairs of dimers—CD3δε, CD3γε, CD3ζζ, and TCR αβ chains—in a 1:1:1:1 ratio [8]; and (4) expression of all the CD3 subunits is required for optimal CD3/TCR signaling involved in T cell activation and ontogeny [24].

Each TCR contains two ε chains, one in each of the heterodimeric CD3δε and CD3γε subunits (Figure 1). The CD3ε chaperones the assembly and folding of all other CD3 subunit proteins, and thus, is essential for CD3/TCR expression [25,26,27]. Furthermore, CD3ε has a pivotal role in T cell activation, since it contains a cryptic proline rich sequence that is exposed on the cytoplasmic tail of the ε chain [28,29,30,31,32]. This conformational change has been shown to be necessary for further downstream signaling leading to T cell activation. This sequence has also been suggested to also play a role in amplifying T cell responses of low affinity T cell cognates [33]. It has also been implicated in initiating the recruitment of LCK that drives the ITAM phosphorylation cascade [34,35]. 

The δ and γ chains are highly homologous and perhaps arose from gene duplication [36]. Both γ and δ chains pair with ε chains, whose sequence is more conserved [13]. Some studies show that the γ and δ chains compete for binding with the ε chain and their homology allows them to pair interchangeably with the TCR chains for assembly [19]. Nevertheless, it is possible that δ and γ CD3 chains play slightly different roles in how they interact with the various subunits of the TCR and perhaps even in how they transmit T cell activation signals [9]. 

Transmembrane regions of all the CD3 subunits and TCR chains are in close association with each other through complementary electrostatic interactions between oppositely charged amino acid residues [8] (Figure 1). The TCR α associates with the heterodimeric CD3 δ-ε subunit whilst the TCR β chain associates with the CD3 γ-ε chains [8,22]. The ζ-ζ homodimer associates with the TCR α chain. Each of the CD3 subunits has acidic residues that are complementary and opposite in charge to the basic residues found in TCR α and β chains. This complementary electrostatic organization keeps the transmembrane coils of each of the TCR/CD3 subunits enrobed into each other, tethering the TCR/CD3 complex firmly into the cell membrane, and allowing it to move within the lipid bilayer as a single, independent functional unit [7,11] (Figure 1). 

In α-β T cells, all the subunits of the TCR/CD3 complex are necessary and required for detection of the TCR complex on the cell surface by antibodies [37]. Several studies have shown that mutations or absence of even one of the CD3 proteins or TCR chains leads to levels of cell surface expression that are not detectable by conventional antibodies [24]. CD3/TCR subunits that are unable to pair with their complementary subunits might get retained in the ER, while incompletely assembled CD3/TCR pseudocomplexes stay sequestered in the ER and are translocated to the cytoplasm where they are targeted for lysosomal degradation [38]. The requirements for TCR expression were also confirmed by several transfection-based studies that tried to reconstitute an artificial TCR/CD3 complex in a mock T cell system in vitro. CD3 and TCR chains were introduced in varying permutations into host COS cells, but surface expression of the native conformation of the CD3/TCR complex was detected only when all of the four subunits were present in the cell [39,40]. This was also demonstrated in dog-pancreas-microsome-based mock T cell assemblies [39].

The ε chain and ζ chains are especially important in chaperoning and instructing the assembly and folding of the CD3 protein complexes [41]. CD3ε seems to have a dominant negative effect for TCR/CD3 expression, most likely because it pairs with both γ and δ chains to form CD3γ-ε and CD3δ-ε, whose expressions are necessary for TCR expression and thymocyte development. Their presence is required for the proper folding of the rest of the CD3 subunits [27]. CD3ɛ^−/−^ mice show complete lack of mature thymocytes and not just a reduced number of T cells as in the case of γ, δ, or ζ deficiency. This effect can be rescued by the reintroduction of the CD3ε transgene. The ζ chain is important for the last stage of assembly. It is the last component that joins the assembly line and is involved in confirming the quaternary structure and surface expression of the TCR [26]. A ζ deficient T cell line fails to express TCR/CD3 on its surface, but the reintroduction of the ζ transgene rescues TCR expression [42].

Whether these CD3 subunits are also present in a similar manner in the γδTCR of γ-δ T cells is yet to be confirmed. In γ-δ T cells, some reports have suggested that the CD3δε subunit is absent, and the TCR chains are flanked by two CD3γε subunits instead [22,43]. Other studies have supported this finding where a genetic deficiency in γ chain expression prevented the formation of γ-δ T cells, whilst a δ chain deficit did not have any effect on γ-δ T cell development [44,45].

Further insights into the role of the CD3 complex are also demonstrated by the study of immunodeficiencies in humans and mice [41,45,46]. CD3 deficiencies present as different gradations of Severe Combined Immunodeficiency Disease (SCID) [47,48]. SCID is associated with T lymphocytopenia that involves a low number or no detectable mature T cells in patients. These T cells may be functionally defective in vitro with hampered T cell activation and mitogenesis. ɛ, ζ, and δ deficiencies are particularly life-threatening and lethal, whilst a γ deficiency seems to be less severe, with some reported partial SCID phenotypes that allowed patients to survive into adulthood. The milder immune insufficiency caused by the γ chain defect suggests a possible hierarchical δ > γ relationship in the development and maturation of T lymphocytes in humans. Whether these differences in thymocyte development and maturation also extend to their roles in T cell activation and ITAM based signaling, and eventually antitumor immune responses, is yet to ascertained.

### 2.2. Signaling Motifs in the CD3 Chains Protein Complex

Another primary and essential function of the CD3 complex involves signal transduction via their cytoplasmic tails which contain ITAMs. ITAMs consist of highly conserved consensus amino acidic sequences arranged in the following motifs: YXXL/I X6-8 YXXL/I [11]. Tyrosine residues located in ITAMs are preferentially phosphorylated by Src protein tyrosine kinase family members such as LCK and FYN and serve as docking sites for other tyrosine kinases such as ZAP70 that continue the signaling cascade [9]. This pathway eventually leads to the production of second messengers (Ca^2+^ and IP_3_) and Ras activation, which induces the activation of transcription factors such as NF-κB, NFAT, and AP-1, the characteristic signature of an activated T cell (Figure 1).

The γ, ε, and δ chains have one ITAM each, whilst the ζ chain has three ITAMs. As a result, each α-β T cell has a total of 10 ITAMs per TCR, 2 each on the CD3δ-ε and CD3γ-ε subunits, and 6 shared between the two ζ chains [10] (Figure 1). What is the purpose of having so many ITAMs in a single TCR? Two models have been proposed to explain this: “redundancy of signaling” and “differential signaling” [23,49]. The redundancy of signaling model suggests that engagement of all ITAMs is not required for T cell activation [23]. Activation of a T cell is determined by engagement of a specific, yet unknown, number of ITAMs (but not all 10 ITAMs) found distributed across all the CD3 subunits. Thus, ITAMs are present in excess numbers to safeguard against the nonactivation of T cells in case the threshold engagement needed for T cell activation is not reached. The differential signaling model proposes that ITAMs in CD3-γ, -δ, -ε, and -ζ chains have distinct functions and control differential activation, proliferation, and effector functions. This model allows for the description of CD3 signaling as a tunable, customizable, and versatile paradigm, where the desired T cell function can be executed by targeting desired ITAMs. 

### 2.3. TCR Triggering

The process in which the TCR/CD3 proteins work together to receive, interpret, and initiate the process of T cell activation is known as T cell triggering [9]. It involves mechanical reception of cognate antigen by the extracellular TCR and subsequent intracellular changes in the CD3 complex and is a prerequisite for T cell activation and the generation of T cell mediated adaptive immunity [9]. The TCR’s organization as an oligomeric, multi-subunit protein complex makes its triggering and sustained signaling a very precise and fine-tuned process that is spatially, temporally, and mechanically regulated and modulated [9,50,51,52,53,54,55,56]. Triggering the TCR is not just dependent on mere interaction of agonistic pMHC or ligand with the TCR, but it is also incumbent upon how, when, and in what context the presentation of the stimulus takes place.

Several models have been proposed to describe TCR triggering—the process of how ligand binding results in an increase in ITAM phosphorylation that allows for subsequent TCR activation. These models may also provide explanations for the exquisite specificity and sensitivity that the TCR exhibits. The main models of TCR triggering are: the Conformational Change model that proposes allosteric regulation of the TCR/CD3 complex [50]; the Kinetic Segregation Model that depends on macromolecular clustering of the TCR for activation [55]; and the Mechanosenor model that implicates the application of anisotropic—direction dependent—force for T cell triggering [51]. Probably all of them are involved to a certain extent in the TCR triggering [57], making TCR engagement and T cell activation a dynamic, multifactorial process.

TCR triggering eventually leads to ITAM phosphorylation, recruitment of ZAP-70, LAT and consequent calcium flux, and downstream activation of transcription factors such as NF-κβ, NFAT, and AP-1 that is characteristic of an activated T cell [58]. Ligand binding seems to induce a conformational change in the cytoplasmic domains of the TCR/CD3 complex that induces release of the cytoplasmic tails of the CD3ε and ζ sequestered by ligation of its basic rich sequences (BRS) to acidic lipids and cholesterol present in the inner leaflet of the plasma membrane [50,56]. Upon release from the plasma membrane, CD3ε can expose its PRS, a proline rich sequence that ligates with the SH-3.1 domain of the NCK adaptor protein [56]. This exposure of the CD3ε PRS is a preactivation state that is the necessary and essential for the phosphorylation of the ITAM tails of CD3ζ as well as CD3ε. A mere 5% of CD3ε species that cannot adopt this conformation can abrogate TCR signaling, making this a functionally dominant pre-requisite for T cell triggering and suggesting cooperative mechanisms of signaling between TCRs [50,56].

In addition to resolving the intrinsic composition of the TCR as described above, structural organization or clustering models of the TCR—where TCRs are hypothesized to be arranged as either dimers and/or oligomers (instead of monomers)—have also been described. Several of these models were developed for explaining the mechanism of action underlying TCR triggering, which can also be described as a function of clustering and association between TCRs on the surface of the T cell. 

Initial studies with soluble ligands clearly showed that monovalent ligands, CD3 antibody-derived F(ab) fragments, or soluble pMHC complexes were unable to stimulate T cells, whereas the same agents, either as a F(ab)2 complete antibody, or di- or multimeric pMHC complexes were able to do so [59,60], indicating the need of physically crosslinking multiple TCRs to induce T cell activation. When bound to a solid surface, monovalent reagents were able to stimulate T cells, likely by forming multivalent arrays on the solid substrate. This conditional activation capacity of monomeric TCR binders is exploited in various antitumor immuno-therapies (see below).

## 3. Strategies to Modulate T Cell Responses Targeting CD3

### 3.1. CD3 Agonistic Therapies to Rescue Function of T Cells

Several strategies have been developed over the years to rescue and boost T cell effector function in the immunotherapeutic treatment of cancerous diseases. Some strategies reinvigorate immunity indirectly by reprogramming the immunosuppressive elements in the tumor microenvironment (TME). Some targets aim to restore metabolic and nutrient balance [61]. HIF1-α inhibitors reduce hypoxia [62]; CD39, CD73, and adenosine receptor blockade prevents adenosine-mediated immunosuppression [63]; and glucose decoys such as 2-deoxyglucose (2-DG) [61] and glucose transport inhibitors [64] restore metabolic and nutrient availability for T cells by preventing excessive glycolysis in the TME. Other drugs block the function of negative immune regulators: anti-CTLA4, anti-PD1/PDL1, anti-TGF-β, or anti-CD47 blocking agents might enhance T cell infiltration in the TME [65].

One of the interventions that may rescue dysfunctional T cells in an immunosuppressive TME consists of providing T cell-activating stimuli in the form of TCR/CD3 agonists. The CD3 protein complex is the signaling subunit of the T cell, and thus, special attention has been paid to developing strategies that could rescue T cell function via targeting the axis where the first signal of T cell activation takes place through the CD3/TCR engagement. These interventions modify function and/or provide stimulatory signals directly to the T cells to rescue their activation potential, effector function, as well as memory formation ability. 

The CD3 subunits are highly desirable targets for cancer immunotherapy due to several advantages that it may afford. The CD3 complex is a favorable target because of its nature as the signaling-machinery-orchestrating subunit of the T cell receptor. Its function, thus, makes it a target accessible for signal modulation and redirection. The CD3 subunits are the nonvariant subunits of the T cell receptor complex and are present on all T cells—both CD4+ and CD8+ T cells and α-β and γ-δ T cells—making them an easily accessible, pan-T cell, off the shelf, universal target. The multimeric nature of the CD3 complex—6 chains of 3 different types of CD3 proteins, 10 ITAMs of 6 different types as well as their modular arrangement as hetero and homodimers—provides a high degree of modularity and several foci of therapeutic intervention where the CD3 agonistic signal can be fine-tuned and customized to exhibit a wide range and scope of regulatory and modulatory effects on the T cell activation axis. Furthermore, targeting the CD3 subunits may provide the advantage of activating the T cell without depending on the context of MHC restriction. Still, not many drugs—immunotherapy or otherwise—have been developed targeting the CD3 subunits; it is a gap in therapy and an unmet medical need that we are yet to explore and to fill. Some of these CD3 modulators, and their pre-clinical and clinical use, have been summarized in Table 1.

Several mechanisms to potentiate T cell activation have been described in the literature. Monoclonal antibodies (mAb) agonistic to the CD3 and TCR subunits, bispecific antibodies and T cell engagers (BiTEs), and modified CD3 antibody fragments directly target and ligate T cells to provide agonistic signals. Adoptive T cell therapies, including CAR T cell therapies and TCR-T cell therapies outsource the T cell activation and expansion process to an ex vivo setting through the isolation of patient-derived or allogeneic T cells followed by ex vivo modification, activation, expansion, and subsequent reinfusion into patients. Other strategies use APC biomimetics to expand T cells ex vivo before adoptive transfer, expanding artificially the number and quality of activated tumor reactive T cells that are available in vivo. In this review, we will focus on those strategies that directly target the CD3/TCR complex in situ—namely, CD3 specific antibodies, their variants, and CD3 aptamers (Table 1).

### 3.2. Anti-CD3 mAbs

In vitro studies performed in the 1980s using anti-CD3 mAbs showed that stimulating human T cells with the anti-CD3 antibodies can have distinctly different outcomes depending upon the mode of stimulation. When presented as immobilized on microbeads, anti-CD3 antibodies induced robust proliferation and activation of T cells, exhibiting a mitogenic effect. On the other hand, under proliferation-inducing conditions, if CD3 antibodies were cross-linked in solution, and presented in their soluble form to T cells, a weaker and even abortive signaling response was generated [85,86]. Thus, these complex T cell activation dynamics were the acting principles used in designing in vivo preclinical studies using the anti-CD3 antibodies that formed the stepping stones to developing the CD3 antibody-based clinical therapies used today.

Further studies in the 1980s using murine anti-CD3 mAbs showed initial efficacy in reducing tumor growth in vivo; paving the way for human CD3 mAb-based therapies [87]. Muromonab, or OKT3 [88], a mAb targeting the human CD3, was discovered in the late 1970s [89], and was touted to be the magic bullet that cures cancer due to its ability to provide agonistic signals to the T cells in an antigen independent, MHC restriction-independent manner. Even though the antibody was highly mitogenic, with a robust ability to induce activation, proliferation, and cytolytic activity of T cells in vitro, the in vivo translation of antibody treatment for cancer immunotherapy was unsuccessful [89]. OKT3 administration in patients induced intense cytokine storm associated with the release of TNF-α and IFN-γ, monocyte activation, and complement cascade. Patients receiving OKT3 suffered from fever, chills, severe fatigue, nausea, and other adverse side effects associated with early T cell activation followed by a stage of anergy or apoptosis Haga poptosis. Although withdrawn from use in 2010, Muromonab, due to its T cell activity curbing properties, was previously widely used as a therapy for treating acute cellular rejection after solid organ transplantation since its approval in 1985 [90]. 

With the development of the mouse anti-CD3 mAb 145-2C11 [69], the therapeutic potential and mechanism of action of CD3 murine agonist antibodies could be further tested in animal models. The use of mouse anti-CD3 mAb surprisingly exerts a potent effect in counteracting autoimmunity in vivo, despite its agonistic activity in vitro. The administration of the antibody in nonobese diabetic mice (NOD) over a period of 5 days induced antigen specific, long-lasting remission of the disease, without affecting the response against allografts. The treatment also prevented the generation of an immune response towards syngeneic pancreatic islet grafts, showing that tolerance can be induced by modulating CD3 activation [91]. These preliminary results initiated further studies using the same antibody for tolerance induction in other autoimmune diseases and autoimmune mediated pathologies.

Anti-CD3 antibodies were also tested in experimental autoimmune encephalomyelitis (EAE) animal models of Multiple Sclerosis (MS). In the Lewis rat EAE model, it was observed that therapy with a nonmitogenic, nonactivating anti-CD3 mAb (G4.18) can reverse established EAE in mice [70]. When tested in the PLP139–151 EAE model in SJL/J mice, results were similar: the anti-CD3 antibody was able to reduce the symptoms of the disease, inducing lower central nervous system (CNS) inflammation associated with decreased Ag-specific T cell proliferation [71]. The G4.18 anti-CD3 Ab leads to the induction of immunotolerance by promoting apoptosis of reactive effector T cells and by hampering T cell trafficking [71]. Additional autoimmune animal models were used to test anti–CD3 treatment efficacy, such as the 2,4,6-Trinitrophenyl-Keyhole Limpet Hemocyanin (TNP-KLH)-induced colitis model of inflammatory bowel disease (IBD) [92] and the collagen-induced arthritis model of rheumatoid arthritis [93]. 

The use of anti-CD3 antibodies also gained popularity for use in transplantation. There are several papers demonstrating the efficacy of several CD3 antibodies, in addition to Muromonab as mentioned above, in the induction of tolerance to allografts [94,95,96]. 

Most of the information that we have related to the induction of tolerance with anti-CD3 antibodies has been from studies conducted using NOD mice that spontaneously develop autoimmune diabetes. Teplizumab (a Fc-receptor-nonbinding humanized mAb specific for CD3) induces human gut-tropic regulatory cells in NOD-humanized mice as well as in patients with type I diabetes. This suggests that the tolerogenic effect of CD3 antibodies is not dependent on Fc engagement (no antibody-dependent cellular cytotoxicity (ADCC) or complement-dependent cytotoxicity (CDC) is triggered) and probably is modulated by a strong TCR/CD3 signal in the absence of costimulation leading to T cell anergy or apoptosis [72]. Some of these CD3 antibodies also seem to induce the expansion of regulatory T cells (immunosuppressive T cells that orchestrated peripheric tolerance) [75,76].

Diabetic patients treated intravenously with Teplizumab improved insulin production and metabolic control in clinical trials [73]. Furthermore, Teplizumab has just been FDA approved for its use in delaying the onset of diabetes [74]. 

In another phase II/III study using the anti-CD3 antibody otelixizumab in type 1 diabetes, more of the patients’ pancreatic β cells were preserved upon treatment, especially for recently diagnosed patients [77]. In patients with severe corticosteroid-refractory ulcerative colitis, the anti-CD3 antibody Visilizumab was proven to be an effective and safe treatment option [78]. Unfortunately, in posterior studies, Visilizumab induced secondary side effects such as cytokine release syndrome and increased the rate of infections in patients, and as a consequence, the clinical development of Visilizumab was ceased. 

In a quest to improve immune tolerance and reduce side effects, the use of OKT3 via oral administration was explored. Preclinical studies showed promising results, and a pilot clinical trial with 15 patients demonstrated that this therapeutic plan was safe and efficacious, inducing improved tolerance associated with higher TGF-β and IL-10 production by dendritic cells whilst reducing Th1 and Th17 based T cell responses [97]. Efficacy and safety of oral CD3 administration was also corroborated in another phase II study involving patients with inflammatory hepatic diseases: chronic hepatitis C infection (HCV) and nonalcoholic steatohepatitis (NASH) [98,99].

Today, CD3-specific agonistic antibodies, despite their proposed and untapped potential in the field of cancer immunotherapy, are mainly used as immunosuppressive drugs to prevent acute rejection in transplants, and for the treatment of autoimmune disorders [64,65], as well as for selective depletion of CD3+ lymphoblastic leukemia populations in vivo [66]. In the field of cancer immunotherapy, the use of CD3 antibodies is limited to the use of variants of the anti-CD3 antibody OKT3 for ex vivo expansion of T cells for adoptive T cell therapy [67,68].

### 3.3. The Importance of Providing CD3-Mediated Signaling In Situ: Bi-Specific T-Cell Engagers (BiTEs)

The agonistic potential of CD3 mAbs in the context of cancer immunotherapy could only be harnessed in the past few years, with the advent of bispecific antibody platforms [100,101]. Bispecific antibodies include many different types of constructs, with bispecific T cell engagers (BiTEs) as their most successful class [102]. BiTEs are made up of two scFvs—fusion proteins of the antigen-binding, variable domains of the heavy and light chains. BiTEs bring together two ScFvs—one specific for CD3, and the other for a tumor-associated antigen, fused together in a single bivalent construct [103,104,105,106]. BiTEs’ mechanism of action focuses on redirecting and guiding T cell effector function in an antitumor manner increasing the number of CD3 engagers in the tumor cell proximity. These synthetic bispecific mAbs simultaneously bind to T cells as well as tumor cells and redirect the cytolytic and effector activity of a primed and activated T cells towards targeting malignant cancer cells, thereby, potentiating the antitumor immune response [103,104,105,106]. 

BiTEs catalyze T cell activation only when they are tethered onto tumor cells using their tumor-binding arm due to the monovalency of the TCR binding arm and their soluble nature. Thus, they are able to precisely focalize their T cell activating power. Tumor cells coated with a certain density of BiTEs facilitate multivalent engagement of the TCR/CD3 complex and can promote activation of the T cell through TCR clusterization, bypassing the need of MHC-restricted activation of the T cell, and thus, representing a therapy that, in principle, is applicable in the same format to many patients. The optimized design of BiTEs renders the CD3 agonistic activity only in the tumor site, improving the antitumor efficacy and reducing the side effects of a systemic agonistic T cell activation [103,104,105,106]. 

ScFvs or single chain variable fragments are fusion proteins consisting of the heavy chain and the variable chain of the antigen binding arm of an antibody. CD3 ScFvs as well as ScFvs specific for several tumor-associated cell surface antigens such as CD19, CD20, CD33, B-cell maturation antigen (BCMA), CD123, and CD38, as well as for tumor antigens identified in hematological malignancies [100,103], have been generated for clinical application [100,103]. Using this arsenal, several bispecific BiTEs have been generated. Of particular interest is Blinatumomab, a CD19-directed CD3 T-cell engager [79,80] that was approved by the FDA in 2014 for the treatment of acute lymphocytic leukemia and consists of two scFvs, one engaging the CD3ε on the T cells, and the other engaging CD19 on B cells. Other BiTEs in clinical trials for the treatment of solid neoplasms target carcinoembryonic antigen (CEA) for nonsmall-cell lung carcinoma (NSCLC), Delta-like Ligand 3 (DLL3) for small cell lung cancer, epidermal growth factor receptor variant iii (EGFRviii) for glioblastoma, epithelial cell adhesion molecule (EpCAM) for NSCLC, human epidermal growth factor receptor 2 (HER-2) for breast cancer, Mucin 16 (MUC16) for ovarian cancer, prostate-specific membrane antigen (PSMA) for prostate cancers, and the somatostatin receptor (SSTR2) for neuroendocrine tumors, among others [101,103]. Thus, BiTEs provide off-the-shelf T cell agonism in a tumor-specific manner in vivo. 

In a similar manner, another type of T cell-directed therapy called immune mobilizing monoclonal T-cell receptors against cancer (ImmTAC) are also gaining popularity in the clinic. Tebentafusp is a novel, bispecific fusion between a gp100 peptide-HLA-A*02:01 specific TCRαβ domain and a CD3 ScFv currently in use for the treatment of uveal melanoma and malignant melanoma [81,82]. Tebentafusp was approved by the FDA in 2022, is the first-in-class of TCR-ScFv fusion proteins that concomitantly bind CD3 on T cells alongside tumor antigens using affinity-enhanced engineered TCRs to potentiate antitumor immune responses. The binding of the TCR arm to HLA-A*02:01-positive uveal melanoma cells and CD3 scFvs provides CD3 agonism, activating polyclonal T cells resulting in the release of inflammatory cytokines and cytolytic proteins and the subsequent clearance of tumor cells. In a randomized phase III clinical trial, previously untreated patients receiving Tebentfusp showed improved overall survival and progression-free survival when compared to therapy with single-agent pembrolizumab, ipilimumab, or dacarbazine, showing its efficacy in the treatment of (HLA)-A*02:01-positive metastatic uveal melanoma patients.

Other antibody-based therapeutics have also shown the ability to activate T cells and protect from tumor progression in murine melanoma models. A Fab-fragment generated from a murine CD3 antibody was shown to copotentiate TCR/CD3 signaling in response to weak pMHC antigens. Cross reactivity of this Fab-fragment to human CD3 as well as its clinical translation potential is yet to be studied [31,107].

### 3.4. Aptamers as a Novel Class of CD3 Modulators

Even though cancer immunotherapy has gained success through the various T cell therapies described above, there is still a need for an economical, mass producible, off-the-shelf, reversible, non-cell-based, pan-cancer CD3 agonistic cancer immunotherapy platform that can serve to enhance and restore T cell-mediated antitumor activity. Aptamers may be one of the candidates with the potential to fill this gap.

Aptamers are DNA- or RNA-based synthetic oligonucleotide drugs [108]. They are single-stranded species that adopt complex three-dimensional conformations that allow them to bind and interact with a wide variety of targets with high affinity and specificity. Hence, they behave like “chemical antibodies” with versatile applications and may be used to bind, block, activate, or modulate the activity of any chosen target [108].

Given their biochemical structure, they are amenable to modifications that allow for: protection from nuclease degradation; modular scaffolding and oligomerization with various cargos, including protein, miRNAs, siRNAs, and other aptamers; and tagging with fluorescent dyes, drugs, toxins, and haptens without losing binding efficiency [109,110,111,112]. Furthermore, aptamer binding efficiency can be increased through the inclusion of novel bases in the aptamer library; excising nonbinding motifs in aptamers via truncation; and by bringing together several binding motifs in one aptamer to augment binding affinity [112,113]. Compared to antibodies, they are small in size, lowly antigenic in vivo since they are not protein-based products and have limited half-life and circulation time in vivo [109,112,114,115,116]. They can be neutralized to reverse their in vivo effects with a universal antidote or with the help of an oligonucleotide complementary to the aptamer sequence [109,112,114,115,116]. They can be chemically synthesized in large quantities via good manufacturing practice (GMP) grade mass production at a relatively low cost and lyophilized for long-term storage [109,112,114,115,116]. Aptamers’ pharmacokinetic/pharmacodynamics profile—low immunogenicity, in vivo reversibility, and lack of sustained persistence—might help to prevent off-target effects [109,112,114,115,116]. Combined with their potential for economical mass manufacturing, aptamers might be ideal for clinical translation and maybe the next therapeutic class of drugs to treat diseases [109,112,114,115,116]. This provides aptamers with customizable, versatile, and translatable modality for in vitro as well as in vivo therapies.

Aptamers are selected through the systematic evolution of ligands using EXponential enrichment (SELEX), which is a structured process of pruning randomness [115,117]. During SELEX, a combinatorial library of millions and millions of unique oligonucleotide strands are sequentially and recursively exposed to the target of choice in iterative rounds of binding. These systematic binding screens organically and serendipitously select discrete, unique species—potential aptamer candidates—that are the best binders with robust affinity and exquisite specificity to the desired target.

Aptamer selection is not biased by the antigenicity of certain epitopes contained within the target protein, as happens in the case of selecting antibodies. Many monoclonal antibodies generated against the same protein might be favored to recognize similar overlapping immunodominant epitopes. For example, many CD3 agonistic antibodies that have been generated recognize the CD3ε chain, perhaps attributed to antigenic bias. In contrast, because of their unique biochemical properties, anti-CD3 aptamers might show a higher range of potential interaction sites with the target proteins and exert a wide variety of allosteric, agonistic, or antagonistic activity on the CD3 complex, providing us with a versatile arsenal of applications for cancer immunotherapy. 

A few aptamers have been described to recognize human/murine T cells. A 2020 study described a DNA aptamer selected against the human CD3ε complex on Jurkat cells using a variant of SELEX called ligand-guided selection (LIGS) [83]. Eluted LIGS libraries obtained through Illumina high-throughput DNA sequencing were analyzed to resolve five DNA aptamers with apparent affinities in the low nM range against human CD3ε. The specificity of the aptamers was validated utilizing multiple strategies, including competitive binding analysis and by binding assays using a CD3 double-knockout Jurkat cell line generated via CRISPR/Cas9, and showed that all five candidate aptamers bound to the same CD3-spcific binding site. Thus, LIGS is a universal platform to identify multiple, highly specific aptamers toward multicomponent receptor proteins in their native state without changing the cell-surface landscape [83]. This aptamer was truncated and dimerized upon which it showed robust binding to human T cells and exerted agonistic activity on the T Cells, inducing expression of CD69, a T cell activation marker in vitro [118].

A 2021 patent describes RNA aptamers selected against recombinant CD3ε/γ and CD3ε/δ proteins, followed by several rounds of selection with human CD3+ Jurkat cells [84]. These aptamers showed binding to T cells and were used in vitro to isolate and enrich T cell populations, but no data on their ability to activate T cells has been provided [84].

An interesting observation to note with the development of human CD3 aptamers is the consistent use of the ever-popular Jurkat cell line in the aptamer selection and characterization process. Even though the Jurkat cell line has been well-characterized and is still widely used in research, it is nevertheless, in its essence, a “model” of human immortalized T cell and, thus, may not completely reflect the intricate and complex dynamics that underlie T cell activation in vivo. Such bias may be overcome by promoting the use of primary peripheral T cells along with endogenous TCR-antigen recall models during aptamer selection. Nevertheless, in vitro studies and humanized mouse model studies have provided us with a few aptamers that have the potential to be CD3 modulators.

Several other T cell immunomodulatory aptamers strategies have been described previously [108,119], including targeting the costimulatory receptors such as 4-1BB, CD28, CD40, ICOS, and OX-40. Bispecific aptamers for targeting T cell activation in situ have also been implemented in several studies [108,119]. Thus, it is feasible also to generate BiTE-like aptamers with bispecific capacity to bind CD3 and a tumor receptor, thus delivering CD3 aptamer engagers to tumor cells.

### 3.5. Targeting CD3 Complex with Small Molecules to Modulate T Cell Activation

Even though the TCR/CD3 complex has been identified as a valuable target in the toolbox used to modulate immune responses, there are only a few small molecules in clinical development to target the function of this receptor. 

One of the first-in-class TCR inhibitors was developed by the Alarcon group [120]. This molecule was designed via virtual screening and combinatorial chemistry. It was aimed to inhibit the interaction between the TCR/CD3 complex and the noncatalytic region of tyrosine kinase (NCK). TCR–pMHC binding induces a conformational change that exposes CD3ε cytoplasmic proline rich sequence (PRS) domains where NCK and LCK induce ITAM phosphorylation leading to T cell activation [121]. The blockade of NCK with the small molecule AX-024 modulates T cell activity by inhibiting ITAM phosphorylation and, thereby, preventing T cell activation. This drug is currently being explored for its use in autoinflammatory diseases such as psoriasis and asthma [120]. 

There are a few other candidate drugs that could be explored to improve CD3 signaling by modulating tyrosine phosphatase activity. LCK is an essential Src kinase that initiates the T cell phosphorylation of the CD3 ITAM, and its activity is tightly regulated by cycles of phosphorylation and dephosphorylation. Phosphorylation of LCK at Y505 keeps the kinase inactive while phosphorylation of Y394 is characteristic of an active kinase. CD45 is probably one of the main phosphatases involved in this process as it can eliminate the phosphate groups from both tyrosines with different efficacies, thus the CD45 expression levels conditions the extent of T cell activation [122]. Moderate expression of CD45 exerts a positive effect on T cell activation by eliminating the phosphate group from Y505. Conversely, a high expression of CD45 on the surface of the T cell leads to further phosphatase activity, removing the more protected phosphate group from Y394. In addition to CD45, there are other phosphatases, such as SHP1, PTPN2, or PTPN22, among others, whose role cannot be underestimated in determining the fate of T lymphocyte activation upon TCR engagement [122]. Many of these phosphatases have become interesting druggable targets in cancer immunotherapy. 

Another option is to intervene in a negative feedback loop that restricts the T cell activation further downstream of the CD3 signaling pathway. Diacylglycerol (DAG) is a second messenger whose phosphorylation is essential for triggering AP-1, NF-κB, and NFAT, the three canonical transcription factors involved in T cell activation. A panel of drugs aimed at modulating the activity of the diacylglycerol (DAG) kinases (DGKs) are in evaluation to improve the T cell activation, with promising preclinical results reported for Ritanserin, a potent serotonin 2A receptor antagonist, and for R59949, which amplified TCR signaling by specific inhibition of DGKα; thereby, amplifying the TCR signaling [123]. 

However, some of these small molecule drugs might exert other undesirable side effects as many of these targets are not exclusively expressed in T cells. However, targeted inhibition of any of these possible modulators using a delivery vehicle such as an antibody or an aptamer to achieve the strategic modulation of the pathway only in the T lymphocytes could optimize the therapeutic window [108]. 

### 3.6. Conclusions

The TCR/CD3 complex determines the specificity of the immune response and is the first signal needed to trigger T cell activation. The development of new approaches to modulate the activity of this complex is very attractive for immunotherapy. Most of the current studies are based on antibodies and chimeric bispecific proteins, but there are also other therapeutic platforms to be explored that could bring new advances in the field such as aptamers, oligonucleotide base ligands that can be used to target new modulatory epitopes in the TCR/CD3 complex, to generate new bispecific molecules, or as delivery agents to target other CD3 immunomodulatory drugs to T cells. The recent advent of CD3-targeted therapies has opened the door to improving the therapeutic outcome of immunological disorders, as highlighted in this review, in the fields of cancers and autoimmunity.

## Figures and Tables

**Table 1:** Selected summary of preclinical and clinical uses of CD3 modulators.

Name	Type of T Cell Modulator	Target	In Vitro Effect	In Vivo Effect
OKT3	Anti-CD3 antibody	Human	Induces activation, proliferation, and cytolytic activity of T cells in vitro [66]	Used to prevent acute rejection in transplants, for the treatment of autoimmune disorders [64,65], and for depleting CD3+ lymphoblastic leukemia populations in vivo [66]. Furthermore, variants of OKT3 are also used to expand T cell adoptive therapy populations ex vivo [67,68].
145-2C11	Anti-CD3 antibody	Mouse	Agonistic activity in vitro [69]	Induces immune tolerance in vivo and tolerance towards syngeneic pancreatic islet grafts in preclinical models of diabetes [69]
G4.18	Anti-CD3 antibody	Mouse	Nonmitogenic, nonactivating in vitro [70]	Induces immunotolerance in vivo in preclinical animal models of Multiple Sclerosis (MS) [70,71]
Teplizumab	Anti-CD3 antibody	Human	NA	Delays onset, reduces activity of autoreactive T cells, and induces T regulatory cells [72,73,74,75,76]
Otelixizumab	Anti-CD3 antibody	Human	NA	Used in the treatment of type 1 diabetes—improves preservation of the β cells mass in the pancreas [77]
Visilizumab	Anti-CD3 antibody	Human	NA	Used in the treatment of severe corticosteroid-refractory ulcerative colitis [78]
Blinatumomab	CD19-directed CD3 T-cell engager	Human	NA	Used in the treatment of acute lymphocytic leukemia [79,80]
Tebentafusp	gp100 peptide-HLA-A*02:01 directed T cell receptor (TCR) CD3 T cell engager (immune mobilizing monoclonal T-cell receptors against cancer (ImmTAC))	Human	NA	Used in the treatment of uveal melanoma and malignant melanoma [81,82]
CD3-specific DNA Aptamer generated via LIGS	human CD3ε complex on Jurkat cells	Human	Robust binding to human T cells and induction of CD69, a T cell activation marker [83]	NA
CD3-specific RNA aptamer	Recombinant human CD3ε/γ and CD3ε/δ subunits	Human	Binding to T cells. They do not show the ability to activate the T cells [84]	NA

## Abbreviation

%	Percentage
°C	Degrees Celsius
<	Less than
>	Greater than
145-2C11	Mouse ati-CD3 antibody
2-DG	2-Deoxy-D-glucose; glucose analog
3D	three dimensional
4-1BB	Costimulatory Receptor
α	alpha
β	beta
δ	delta
ε	epsilon
γ	gamma
ζ	zeta
αβ	alpha-beta TCR chains
γδ	gamma-delta TCR chains
δε	delta-epsilon CD3 subunit
γε	gamma-epsilon CD3 subunit
ζζ	zeta-zeta CD3 subunit
ADCC	Antibody-dependent cellular cytotoxicity
AP-1	Activator protein; key transcription factor involved in T cell activation
APC	Antigen Presenting Cell
aPD-1	Anti- PD-1 antibody; immune checkpoint blockade
aPDL-1	Anti- PD-L1 antibody; immune checkpoint blockade
AX-024	T cell inhibitor
BCMA	B-cell maturation antigen; Tumor associated antigen in hematological
BCR	B Cell Receptor, expressed on B cells
BiTE	Bispecific T Cell Engager; CD3Ab-TAA bispecific antibody
BRS	Basic-Rich Stretch; signaling motif in CD3/TCR subunits
Ca2+	Calcium ions; released upon T cell activation
CAR T cells	Chimeric Antigen Receptor T cells; Modified T Cell based cancer immunotherapy
CD123	Tumor associated antigen in hematological cancers
CD19	Tumor associated antigen in hematological cancers
CD20	Tumor associated antigen in hematological cancers
CD27	Tumor associated antigen in hematological cancers
CD28	Costimulatory Receptor
CD3	Cd3 signalling subunit of the CD3-TCR complex on T cells
CD3+	CD3 expressing
CD33	Tumor associated antigen in hematological cancers
CD38	Tumor associated antigen in hematological cancers
CD39	Enzyme that converts ATP to ADP; ATP/adenosine pathway
CD3ɛ^−/−^	CD3 knock out
CD3-TCR	CD3-T Cell Receptor complex expressed on T Cells, composed of 1:1:1:1 ratio TCRαβ:CD3γε:CD3δε:CD3ζζ subunits
CD3δ	CD3 delta
CD3ε	CD3 epsilon
CD3γ	CD3 gamma
CD3ζ	CD3 zeta
CD3γε	Gamma-Epsilon heterodimeric subunit of the TCR/CD3 receptor complex
CD3δε	Delta-Epsilon heterodimeric subunit of the TCR/CD3 receptor complex
CD3ζζ	Zeta-Zeta homodimeric subunit of the TCR/CD3 receptor complex
CD4+	CD4+ Helper T cell
CD40	Costimulatory Receptor
CD43	Protein tyrosine phosphatase that favors dephosphrylation of ITAMs
CD45	Protein tyrosine phosphatase that favors dephosphrylation of ITAMs
CD47	“Don’t eat me” signal expressed on tumor cells to prevent phagocytosis by macrophages
CD69	Early T cell activation marker
CD73	Enzyme that converts AMP to Adenosine; ATP/adenosine pathway
CD79αβ	Signalling subunit of the B Cell Receptor
CD8+	CD8+ Cytotoxic T cell
CDC	complement-dependent cytotoxicity
CDRs	Complementarity-determining regions on T cells; involved in pMHC binding
CEA	Carcinoembryonic antigen; a type of tumor associated antigen
CNS	Central Nervous System
CRISPR/Cas9	clustered regularly interspaced short palindromic repeats/CRISPR-associated protein 9; genomic engineering technique
CTLA-4	Negative regulator of the immune response; Exhaustion marker on T cells
DAG	Diacylglycerol kinase involved in T cell activation signaling
DGKα	Diacylglycerol Kinase Alpha
DLL3	Delta-like ligand 3; tumor associated antigen in neuroendocrine tumors
DNA	Deoxyribonucleic acid; Double stranded nucleic acid composed of the bases A, T, G and C
EAE	Experimental autoimmune encephalomyelitis
EGFRviii	Epidermal growth factor receptor variant III; tumor associated antigen in glioblastoma
EM	Electron Microscopy
EpCAM	Epithelial Cell Adhesion Molecule; tumor associated antigen
Fab	Antigen-binding fragment on antibody
Fc	Fragment crystallizable; Antigen non-binding fragment on antibody
FDA	U.S. Food and Drug Administration
FYN	src superfamily protein tyrosine kinases involved in ITAM phosphorylation
G4.18	Anti-CD3 antibody
GMP	Good manufacturing practice; production quality regulations for clinical products and therapies
gp100	Cognate peptide for PMEL T cells
HCV	Hepatitis C Virus
HER-2	Human epidermal growth factor receptor 2; tumor associated antigen in breast cancers
HIF1-α	Hypoxia-inducible factor 1-alpha; key transcription factor that drives hypoxia
HLA	Human leukocyte antigens
IBD	inflammatory bowel disease
ICB	Immune Checkpoint Blocakde
ICOS	Inducible T-cell COStimulator; Costimulatory Receptor
IFN-γ	Interferon Gamma; T cell activation cytokine
IL-10	Interleukin-10; anti-inflammatory cytokine
ImmTAC	Immune mobilizing monoclonal T-cell receptors against cancer
IP3	Inositol trisphosphate
ITAM	Immunoreceptor tyrosine-based activation motif; involved in activation of T cells
lL-2	Iinterleukin-2; T cell activation cytokine
LCK	src superfamily protein tyrosine kinases involved in ITAM phosphorylation
LIGS	Ligand-Guided Selection; a type of modified SELEX
mAb	Monoclonal antibody
MHC	Major Histocompatibility Complex; proteins involved in self-discrimination
miRNAs	microRNA; non-coding ssRNA molecule
MS	Multiple Sclerosis
MUC16	Mucin 16; tumor associated antigen
NASH	nonalcoholic steatohepatitis
NCK	Non-catalytic region of tyrosine kinase; second messenger in T cell signalling
NFAT	Nuclear factor of activated T-cells; key transcription factor involved in T cell activation
NF-κB	Nuclear Factor kappa-light-chain-enhancer of activated B cells; key transcription factor involved in T cell activation
nM	Nanomolar
NOD	Nucleotide oligomerization domain
NSCLC	Non-small-cell lung carcinoma
OKT3	Human Anti-CD3 antibody
OX-40	Costimulatory Receptor
PD-1	Programmed cell death protein 1; negative regulator of the immune response; Exhaustion marker on T cells
PDL-1	Programmed death-ligand 1; negative regulator of the immune response
PLC	Phospholipase C; second messenger in T cell activation signaling
PLP139–151 EAE	PLP139–151 peptide-induced Experimental autoimmune encephalomyelitis
pMHC	Peptide-Major Histocompatibility Complex; cognate ligand for the T cell Receptor
PRS	Proline-Rich Stretch; signaling motif in CD3/TCR subunits
PSMA	Prostate-Specific Membrane Antigen; tumor associated antigen in prostate cancer
pSMAC	Peripheral supramolecular activation complex; macromolecular structure of TCRs in activated T cells
PTPN2	Protein tyrosine phosphatase non-receptor type 2
PTPN22	Protein tyrosine phosphatase non-receptor type 22
RAG1/2	Recombination-activating gene 1/2
Ras	Rat sarcoma virus protein; small GTPase
RNA	Riboxynucleic Acid; single stranded oligonucleotide containing the bases Adenine, Guanine, Uracil and Cytosine.
scFvs	Single Chain Variable Fragment; fusion protein of heavy and variable chains of the antigen binding arm of an antibody
SCID	Severe combined immunodeficiency
SELEX	Systematic Evolution of Ligands by Exponential Evolution; the process through which aptamers are identified
SH.3	Src Homology 3 (SH3) domains; signaling motif present in protein tyrosine kinases
SHP1	Src homology region 2 domain-containing phosphatase-1
siRNAs	Small interfering RNA; non-coding RNA used to silence genes
SJL/J	Swiss Jim Lambert EAE mice
Src	src superfamily protein tyrosine kinases involved in ITAM phosphorylation
SSTR2	Somatostatin receptor 2; Tumor associated antigen in pancreatic cancer
TCR	T Cell Receptor; expressed on T cells
TCR-CD3	CD3-T Cell Receptor complex expressed on T Cells, composed of 1:1:1:1 ratio TCRαβ:CD3γε:CD3δε:CD3ζζ subunits
TCR α-β	alpha beta T Cell
TCR-scFv	fusion protein containing a TCR and a single chain variable fragment
TCRα	alpha chain of the TCR
TCRβ	beta chain of the TCR
